# Palmitate-Induced β-Cell Dysfunction Is Associated with Excessive NO Production and Is Reversed by Thiazolidinedione-Mediated Inhibition of GPR40 Transduction Mechanisms

**DOI:** 10.1371/journal.pone.0002182

**Published:** 2008-05-14

**Authors:** Sandra Meidute Abaraviciene, Ingmar Lundquist, Juris Galvanovskis, Erik Flodgren, Björn Olde, Albert Salehi

**Affiliations:** 1 Department of Clinical Science, Division of Endocrine Pharmacology, the Malmö University Hospital (UMAS), Malmö, Sweden; 2 Department of Experimental Medical Science, University of Lund, Lund, Sweden; Mayo Clinic College of Medicine, United States of America

## Abstract

**Background:**

Type 2 diabetes often displays hyperlipidemia. We examined palmitate effects on pancreatic islet function in relation to FFA receptor GPR40, NO generation, insulin release, and the PPARγ agonistic thiazolidinedione, rosiglitazone.

**Principal Findings:**

Rosiglitazone suppressed acute palmitate-stimulated GPR40-transduced PI hydrolysis in HEK293 cells and insulin release from MIN6c cells and mouse islets. Culturing islets 24 h with palmitate at 5 mmol/l glucose induced β-cell iNOS expression as revealed by confocal microscopy and increased the activities of ncNOS and iNOS associated with suppression of glucose-stimulated insulin response. Rosiglitazone reversed these effects. The expression of iNOS after high-glucose culturing was unaffected by rosiglitazone. Downregulation of GPR40 by antisense treatment abrogated GPR40 expression and suppressed palmitate-induced iNOS activity and insulin release.

**Conclusion:**

We conclude that, in addition to mediating acute FFA-stimulated insulin release, GPR40 is an important regulator of iNOS expression and dysfunctional insulin release during long-term exposure to FFA. The adverse effects of palmitate were counteracted by rosiglitazone at GPR40, suggesting that thiazolidinediones are beneficial for β-cell function in hyperlipidemic type 2 diabetes.

## Introduction

The nutrients glucose and free fatty acids (FFA) are known to have a great impact on the function of pancreatic β-cells [1], [2], [3]. Although glucose is the major stimulus for insulin secretion, its effects are highly modulated by FFA. Insulin secretion might thus be acutely amplified or chronically inhibited by FFA-derived signals [1], [2], [3]. Although interaction between FFA and β-cells plays an important role in insulin secretion, the intimate targets responsible for FFA actions on β-cells are under debate and FFA and cytokines have been claimed to induce β-cell apoptosis by different mechanisms [3]. The acute stimulatory effects have been linked to the action of long chain acyl-CoA molecules on a variety of metabolic sites involved in the insulin secretory pathways [1], [2], [3]. This concept has recently been challenged since the stimulatory action of FFA on insulin secretion, at least in part, was shown to be mediated through a membrane-bound FFA receptor, the G protein-coupled receptor 40 (GPR40) [4], [5], [6]. Notably, the peroxisome proliferator-activated receptor γ (PPARγ), a member of the nuclear receptor superfamily, is involved in islet FFA metabolism. PPARγ is modulated by *e.g.* prostaglandin J2, leukotrine B4 and by a number of recently developed synthetic agents (thiazolidinediones) like rosiglitazone (ROZ) [7], [8], [9], [10]. Since FFA are involved in developing insulin resistance, synthetic agonists of PPARγ have been used clinically to improve glucose tolerance by enhancing insulin sensitivity of adipocytes to suppress lipolysis thus reducing the metabolic burden to liver and muscle that in turn improves glucose homeostasis [7], [8], [9], [10].

Since we have shown that long-term intralipid infusion in rats is accompanied by expression of inducible nitric oxide synthase (iNOS) in pancreatic islets [11], [12], [13], and since excessive NO generation derived from both iNOS and neuronal constitutive NOS (ncNOS) seems involved in impairment of glucose-stimulated insulin release and β-cell dysfunction [14], [15], [16], [17], [18], [19], [20], we found it essential to explore in more detail the effects of FFA on pancreatic islet function. Hence the aim of the present investigation was to study both acute and especially long-term effects of palmitate and its interaction with the PPARγ agonist ROZ on the activities of islet NOS isoenzymes in relation to GPR40 and insulin secretion and thus to further elucidate whether the thiazolidinedione drugs would be of possible therapeutic value for the function of the β-cell in dyslipidemic type 2 diabetes.

## Results

### Acute effects of palmitate and ROZ on PI hydrolysis in GPR40-transfected HEK293 cells and their interaction with PI hydrolysis and insulin release in MIN6c4 cells, as well as palmitate-induced effects on islet NOS activities and effects of ROZ and diazoxide on insulin release from isolated islets

**Figure 1:**
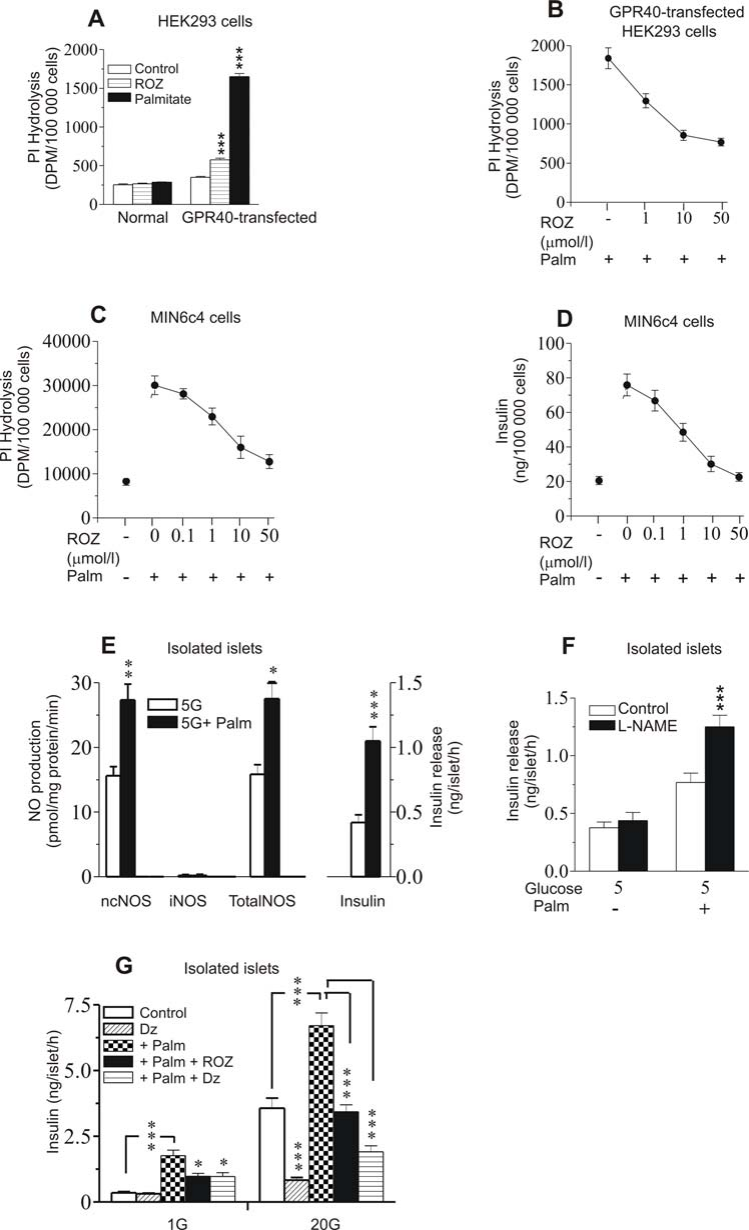
Short-time effects of palmitate and rosiglitazone (ROZ) on phosphatidyl inositol (PI) hydrolysis, in HEK293 and MIN6c4 cells, insulin release in MIN6c4 cells as well as NO production and insulin release in isolated islets. (A) PI hydrolysis in nontransfected and GPR40-transfected HEK293 cells in response to palmitate (1 mmol/l) and ROZ (50 μmol/l) (n = 8–12) and (B); dose-dependent effect of ROZ on palmitate-induced PI hydrolysis (n = 8-12). (C, D) PI hydrolysis and insulin release in MIN6c4 cells in response to palmitate (1 mmol/l)±ROZ at different concentrations (n = 6). (E) NO production from neuronal constitutive nitric oxide synthase (ncNOS), inducible NOS (iNOS) and total NOS as well as insulin release in response to palmitate (1 mmol/l) after 60 min incubation of freshly isolated mouse islets at 5 mmol/l glucose (5G) (n = 4). (F) Effect of the NOS inhibitor L-NAME on insulin release induced by palmitate (1 mmol/l) in the presence of 5 mmol/l glucose (n = 8). (G) Effects of palmitate±ROZ or diazoxide on insulin release from freshly isolated mouse islets incubated at low (1 mmol/l) or high (20 mmol/l) glucose for 60 min. The concentrations of the different test agent were; palmitate (1 mmol/l), ROZ (1 μmol/l), diazoxide (dz) (250 μmol/l) (n = 8-12). Values are mean±s.e.m for. ** p<0.01; *** p<0.001.

We first tested the acute action-interaction of palmitate in relation to ROZ on PI hydrolysis in HEK293 cells transiently expressed with mouse GPR40. HEK293 cells do not express endogenous GPR40 [21] and is well suited to explore the immediate response to GPR40 ligands after transient expression of the receptor. **Fig. 1A** shows that PI hydrolysis after 30 min incubation of GPR40-transfected HEK293 cells in presence of 1 mmol/l palmitate is highly increased compared with nontransfected controls and that ROZ by itself has a significant agonistic action. **Fig. 1B**, on the other hand, shows that ROZ has an inhibitory action on the palmitate-stimulated PI hydrolysis in transfected cells.

Next we used MIN6c4 insulinoma cells, known to endogenously express GPR40 [21] to study whether ROZ interacts with the reported FFA-stimulated activation of PLC *via* GPR40 in insulin-producing cells. MIN6c4 cells were challenged with palmitate for 30 min in absence or presence of ROZ, and PI hydrolysis was analyzed. **Fig. 1C and D**, shows that palmitate-stimulated PI hydrolysis and insulin release in fact were dose-dependently suppressed by ROZ.

To test possible involvement of NOS enzyme activities in relation to palmitate-induced insulin release in acute experiments with primary β-cells, freshly isolated islets were incubated for 60 min. **Fig. 1E** shows basal and acute palmitate-modulated NOS activities and insulin release at 5 mmol/l glucose. ncNOS activity was increased but iNOS was not detectable. Notably, a restraining action of palmitate-induced insulin release through ncNOS-derived NO was revealed by showing a greatly amplified insulin release (**Fig. 1F**) in the presence of the ncNOS inhibitor L-NAME. Hence ncNOS-generated NO abrogated a major part of the palmitate-induced insulin release. Finally, **Fig. 1G** shows that the acute amplifying effect of palmitate on glucose-stimulated insulin release was greatly suppressed by ROZ. In comparison the K_ATP_-channel opener diazoxide was even more efficient (**Fig. 1G**). A similar, although not identical pattern was seen for palmitate-induced insulin release at low glucose (**Fig. 1G**).

### Pattern of insulin secretion and islet insulin content during long-term culturing with palmitate or glucose in the absence or presence of ROZ or diazoxide

**Figure 2:**
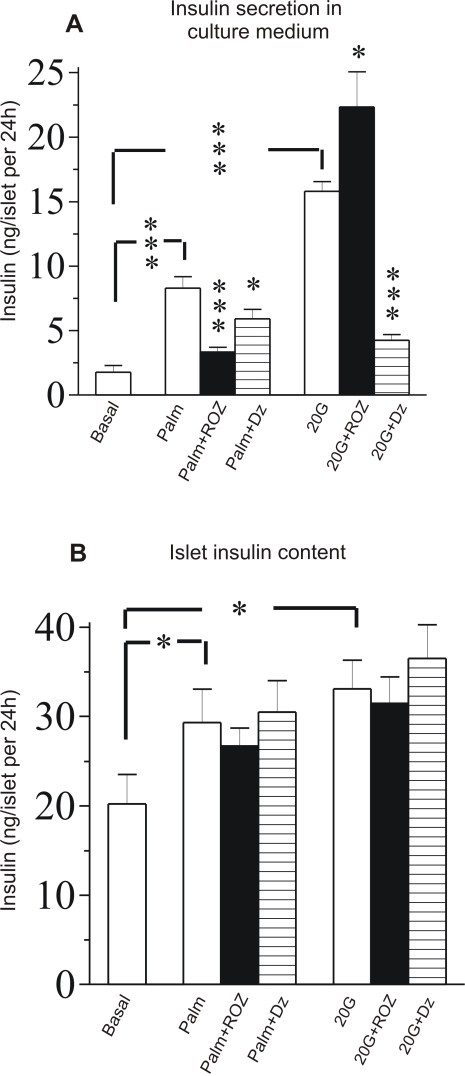
Insulin secretion and islet insulin content after culturing with palmitate or high glucose. Insulin secretion into culture medium (A) and islet insulin content (B) from isolated islets cultured for 24 h at a basal glucose concentration of 5 mmol/l (5G), 5G+palmitate (1 mmol/l) (Palm), 5G+palmitate+rosiglitazone (1 μmol/l) (Palm+ROZ) or 5G+palmitate+diazoxide (250 μmol/l) (Palm+Dz) as well as at high glucose concentration (20 mmol/l) (20G), 20G+rosiglitazone (1 μmol/l) (20G+ROZ) or 20G+diazoxide (250 μmol/l) (20G+Dz). The means±s.e.m for 10-12 batches of islets in each group are shown. Asterisks denote probability level of random difference. * P<0.05; ***P<0.001.

Long-term experiments with palmitate or glucose were performed to study effects of ROZ or for comparison, diazoxide, on insulin secretion and insulin content in islets cultured for 24 h in absence or presence of either agent. **Fig. 2** shows that both palmitate and a high concentration of glucose (20 mmol/l) not only increased insulin release into the culture medium (**Fig. 2A**) but also slightly increased islet insulin content (**Fig. 2B**). ROZ suppressed insulin secretion during culture with palmitate, while slightly amplifying insulin secretion in presence of high glucose (**Fig. 2A**). Diazoxide suppressed insulin secretion during culture with high glucose and only marginally inhibited it in presence of palmitate. ROZ or diazoxide did not affect islet insulin content (**Fig. 2B**).

### Effects of islet culturing with palmitate in presence and absence of ROZ or diazoxide on islet NOS activities and glucose-stimulated insulin secretion

**Figure 3:**
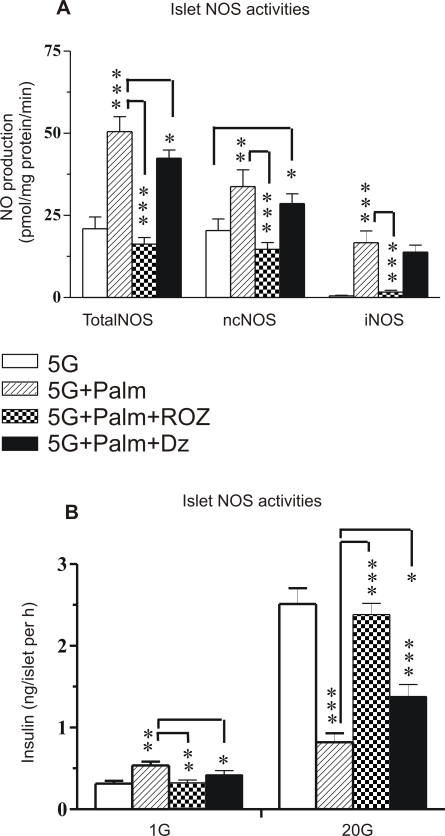
Islet NO generation from neuronal constitutive nitric oxide synthase (ncNOS), inducible NOS (iNOS), and total NOS after culturing with palmitate for 24 h as well as insulin release at basal and high glucose after a subsequent incubation. (A) Total NOS as well as ncNOS and iNOS activities in isolated islets cultured at a basal glucose concentration of 5 mmol/l (5G), 5G+palmitate (1 mmol/l) (Palm), 5G+palmitate+rosiglitazone (1 μmol/l) (Palm+ROZ) or 5G+palmitate+diazoxide (250 μmol/l) (Palm+Dz) (n = 8). (B) Insulin release at low (1 mmol/l) glucose or stimulated by high (20 mmol/l) glucose from islets incubated for 60 min after culturing and washing. The means±s.e.m for 10-12 batches of islets in each group are shown. Asterisks denote probability level of random difference. * P<0.05; ** P<0.01; ***P<0.001.

Islets cultured for 24 h at 5 mmol/l glucose (basal) or 5 mmol/l glucose+palmitate in absence or presence of ROZ or diazoxide were analyzed for ncNOS and iNOS activities. **Fig. 3A** shows that palmitate did not only induce a marked increase in ncNOS activity but also an exclusive activity of iNOS. The stimulatory effect of palmitate on ncNOS and iNOS activities was markedly attenuated when ROZ was present during the culture period. Diazoxide only slightly influenced the stimulatory effect of palmitate on NOS activities (**Fig. 3A**).

A major part of the cultured islets from the same culturing batches were thereafter washed, preincubated in 1 mmol/l glucose for 30 min and incubated at 1 or 20 mmol/l glucose for 60 min. **Fig. 3B** illustrates that insulin release was slightly increased at low glucose when islets had been cultured with palmitate but not when ROZ or diazoxide was present. Conversely, glucose-stimulated insulin release was markedly attenuated after culturing with palmitate. This suppressive effect by palmitate was completely reversed when ROZ was present during the culture period but only slightly so when diazoxide was present.

### Confocal microscopy

**Figure 4:**
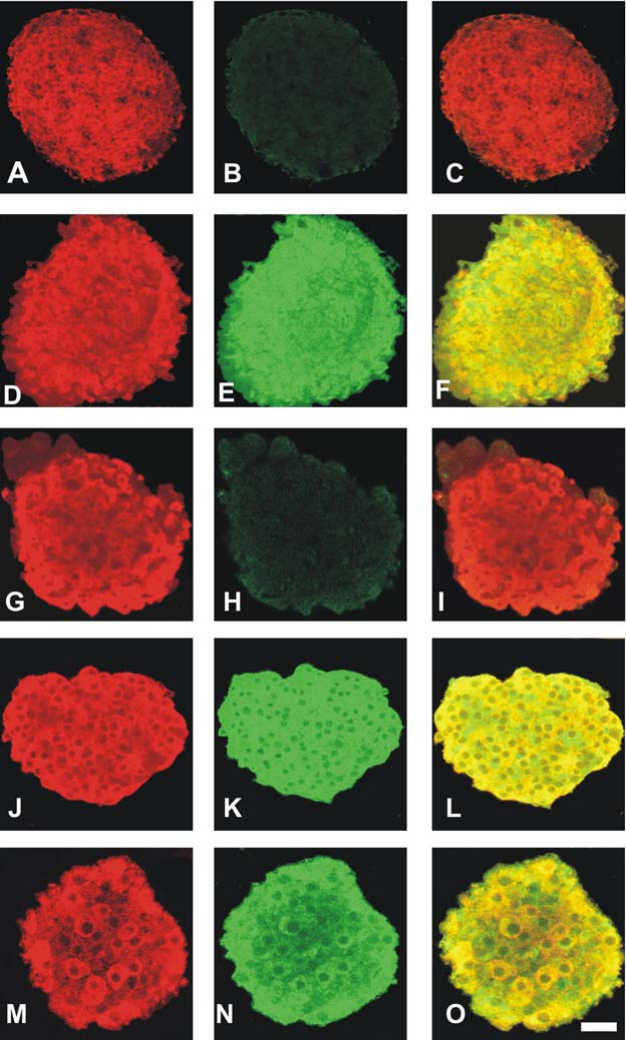
Confocal microscopy of mouse islets. Isolated islets were cultured for 24 h at a basal glucose concentration of 5 mmol/l (A–C); 5G+palmitate (1 mmol/l) (D–F); 5G+palmitate+rosiglitazone (1 μmol/l) (G–I) or 20G (J–L) and 20G+rosiglitazone (1 μmol/l) (M-O). After the culturing period the islets were double-immunolabelled for insulin (appears as red) (A, D, G, J and M) and iNOS (appears as green) (B, E, H, K and N) and analyzed by confocal microscopy. Co-localization of insulin/iNOS is seen as a yellowish fluorescence (C, F, I, L and O). Fluorescence intensity data of iNOS (green) were normalized to 100% as measured in E (5G+palmitate) and gave the following results (n = 12). B = 1.50±0.75; E = 100.6±2.52; H = 4.33±1.50; K = 99.0±2.96; N = 106.1±3.22.

The cellular distribution of iNOS protein was examined with confocal microscopy. **Fig. 4** shows that after culture with palmitate (D–F) or high glucose (J–L) iNOS immunoreactivity was expressed in most islet cells, which also displayed insulin immunoreactivity. No iNOS immunoreactivity was detected in islets cultured at basal glucose (A–C). Addition of ROZ to culture medium suppressed palmitate-induced iNOS expression in β-cells (G–I), whereas glucose-induced expression of iNOS induced by high glucose was not affected (M–O).

### Suppression of GPR40 by antisense M40 in cultured pancreatic islets abrogated palmitate-induced expression of iNOS and suppressed the activities of ncNOS and iNOS as well as insulin secretion

**Figure 5:**
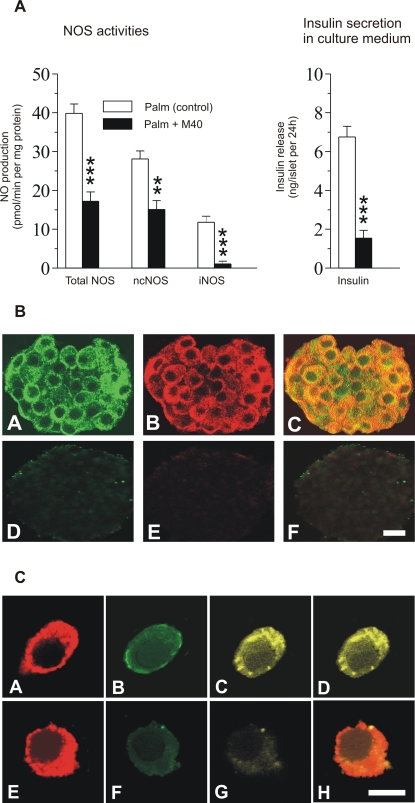
Islet NOS activities, insulin secretion, and expression of GPR40 and iNOS after treatment with palmitate and GPR40 antisense. Isolated islets were pretreated for 24 h with either the M40 antisense morpholino or a non-specific random sequence morpholino (control). The islets were incubated for 30 min in absence or presence of palmitate and then cultured with palmitate±M40 for a further 24 h. (A) M40 caused a marked suppression of palmitate-induced iNOS and ncNOS activities as well as a reduced insulin release (black bars). The results from the control morpholino are indicated by white bars. Values are mean±s.e.m for 4 different experiments performed at different occasions. ** p<0.01; *** p<0.001. (B) Expression of GPR40 (green) and iNOS (red) in islets cultured with palmitate (A-C) or palmitate+M40 (D-F). A and D = GPR40; B and E = iNOS, C and F = overlay. Bar indicates 5 μm. (C) Immunostaining and confocal images of formaldehyde-fixed β-cells. The expression pattern of insulin (red), GPR40 (green) and iNOS (yellow) from dispersed β-cells cultured with palmitate is shown. A = insulin, B = GPR40, C = iNOS and D = overlay. E–H show absence of expression of GPR40 (F) and iNOS (G) after M40 treatment. E = insulin, H = overlay. Bar indicates 5 μm.

To explore whether GPR40 is involved in both palmitate-induced expression of islet iNOS and palmitate stimulation of insulin release during culture with 5 mmol/l glucose we used an antisense (M40) targeting the sequence important for the GPR40 transcript in islets. After culturing, islets were thoroughly washed and processed for the measurement of ncNOS and iNOS activities, insulin release into medium, and detection of iNOS protein. **Fig. 5A** shows abrogation of palmitate-induced iNOS activity, reduction of ncNOS activity, and inhibition of insulin secretion into culture medium in M40-treated islets. Confocal microscopy showed that palmitate-induced iNOS expression was colocalized with GPR40 (**Fig. 5B, A**–C) and abrogated together with GPR40 expression after M40 treatment (D–F). The colocalization of insulin, GPR40 and iNOS is shown in single β-cells (**Fig. 5C**) (A–D). The loss of GPR40 and iNOS proteins after M40 treatment is also shown (E–H).

## Discussion

It is known that FFA are positive modulators of insulin secretion in short-time perspective but become toxic to β-cells when chronically present in elevated levels (lipotoxicity) [1], [2], [3], [8], [9], [10]. Thus prolonged exposure of β-cells to FFA leads to increased basal but suppressed glucose-stimulated insulin secretion [1], [2], [8], [9], [10]. Since many type 2 diabetic patients exhibit elevated plasma levels of FFA and/or increased glucose levels, the underlying mechanisms for the deleterious action of long-term elevation of FFA on β-cells and glucose-induced insulin release would be highly important to elucidate. The present results favor the view that the adverse effects of chronic exposure to FFA is exerted mainly through the newly discovered FFA receptor GPR40 by inducing expression and activity of β-cell iNOS, a highly increased NO generation, and suppression of glucose-stimulated insulin release. Moreover, we show that this deleterious action of FFA on the β-cells is counteracted by the thiazolidinedione drug ROZ, which inhibits GPR40 transduction mechanisms and thus abrogates iNOS expression and in addition restores glucose-stimulated insulin release.

In our initial acute experiments we verified the reported acute activation of PLC and PI hydrolysis by GPR40-mediated stimulation of FFA [5] and now show that this effect when induced by palmitate was inhibited by ROZ in HEK293 cells transiently transfected with GPR40 and in MIN6c4 insulin-producing cells with endogenously expressed GPR40, where ROZ was found to suppress palmitate-stimulated insulin release. These results appear to contradict earlier reports [21] suggesting ROZ to be a GPR40 agonist. However, the previous reports [21], [22] were based on recombinant cell systems with high receptor densities. The most likely explanation is that ROZ is a partial agonist with high affinity and relatively low efficacy, and thus being more dependent on receptor density. We show now that ROZ served as a potent GPR40 inhibitor of palmitate-induced PI hydrolysis and that ROZ dose-dependently suppressed palmitate-stimulated PI hydrolysis in GPR40-transfected HEK293 cells as well as PI hydrolysis and insulin release in MIN6c4 cells. Moreover, we show that acute palmitate-stimulating effects on insulin release were associated with increased ncNOS-derived NO generation, the inhibition of which by the ncNOS inhibitor L-NAME resulted in marked amplification of palmitate-induced insulin response. This acute restraining action by ncNOS-derived NO on palmitate-induced insulin release thus agrees with our previous observations on acute glucose-induced insulin release [17], [18], [19], [20].

We have postulated and presented suggestive data showing that ncNOS-derived NO is rapidly stimulated by glucose and exerts an acute negative feedback action on glucose-induced insulin release [18]. This effect is elicited within minutes and probably involved in creating the nadir separating first and second phase insulin response. This is in accordance with the notion that part of the ncNOS protein is confined to the insulin secretory granules [23]. We also found that the Ca^2+^/calmodulin-dependent ncNOS activity was markedly inhibited by the ATP-sensitive K^+^ channel opener diazoxide [18]. In contrast to the rapid action of glucose on ncNOS activity, there was no effect by glucose on iNOS expression until after ∼1 h of exposure to high glucose *in vitro* and *in vivo* [18], [20]. The Ca^2+^/calmodulin-independent iNOS activity was not inhibited by diazoxide [18] and iNOS-derived NO reportedly exerts cytotoxic and apoptotic effects in β-cells [16], [24]. Importantly, however, in contrast to glucose, the present data show that acute exposure to palmitate did not induce iNOS activity after short-time (∼1 h) incubation suggesting distinct time-dependent mechanisms for palmitate *vs* glucose in iNOS induction and activity. Finally, the acute amplifying effect by palmitate on glucose-stimulated insulin release, which reportedly is linked to activation of GPR40 [5], [6], [25], we now found to be suppressed by ROZ, while the glucose-stimulated part of the release process was unaffected.

We have shown earlier that glucose-stimulated insulin release is disrupted in islets from long-term lipid-infused rats whose islets displayed increased NO production due to induction of iNOS expression [11], [12], [13]. Since we repeatedly found [17], [18], [19], [20] that increased islet NO production in presence of high glucose inhibits insulin release and most likely is taking part in a process of glucotoxicity we are now inclined to ascribe increased iNOS-derived NO in islets cultured with palmitate as an important factor in FFA-induced lipotoxicity. Although there seems to be distinct differences in induction and prevention of iNOS expression and activity elicited by glucose *vs* FFA, increased islet iNOS-derived NO production might be a common denominator in both glucotoxicity and lipotoxicity.

Since ROZ, although debated, has been suggested to be an alternative drug in long-term therapy of type 2 diabetes we were particularly interested in the action of ROZ on long-term effects of FFA, as represented by palmitate, on islet activation of ncNOS and iNOS in relation to glucose-stimulated insulin release. After culturing with palmitate at basal glucose concentration insulin release into medium was enhanced 3-fold compared with basal glucose alone, while islets cultured at high glucose displayed almost 7-fold increase in insulin. Diazoxide abrogated this glucose-induced insulin response, whereas palmitate-induced insulin release was only slightly reduced, suggesting K^+^_ATP_ channel-dependent Ca^2+^ influx mechanisms over time being less important for palmitate-stimulated insulin secretion during this long-term culturing compared with acute effects and possibly more influenced by intracellular Ca^2+^ perturbations [25]. Importantly, during culturing ROZ markedly reduced palmitate-induced but slightly increased glucose-induced insulin secretion into medium. These differences did not depend on islet insulin content, but are likely related to partially differential effects on insulin secretory mechanisms. PPARγ receptors in β-cells show reportedly a comparatively low expression [8] and hence other effects than directly on these receptors might also play a role in the effects of ROZ on insulin release as previously have been suggested [26]. Studies in models of type 2 diabetes have shown that thiazolidinediones enhance β-cell function by mobilizing fat out of the cells [27]. Moreover, it was very recently shown [28] that toxic accumulation of cholesterol in β-cells and associated defects in insulin release could be restored by ROZ through activation of the cholesterol efflux transporter Abca 1, which is upregulated by PPARγ activation. Hence, thiazolidinediones exert diverse beneficial effects on β-cell function as recently has been extensively discussed [9]. Now we show that islets cultured with palmitate displayed marked expression and activity of β-cell iNOS, increased ncNOS activity and a greatly reduced insulin response following a 60 min glucose challenge. After culturing with palmitate+ROZ, however, the increased NO production was abrogated and glucose-stimulated insulin release restored to normal suggesting that ROZ has a direct inhibitory effect on β-cell GPR40. Conversely, after culturing in high glucose+ROZ confocal microscopy showed no effect by ROZ on β-cell iNOS expression. These findings suggest, but do not definitely prove, that iNOS expression and activity in β-cells is regulated through different or partially different mechanisms after long-term exposure to palmitate *vs* glucose. It should be noted that when ROZ was replaced by diazoxide during palmitate culturing iNOS activity was unaffected and ncNOS activity and glucose-stimulated insulin release were less suppressed than after palmitate+ROZ. Attenuation of insulin release by K_ATP_ channel openers like diazoxide is ascribed to β-cell hyperpolarization, thereby providing β-cell rest. Notably, our present data suggest that such a protective effect on glucose-stimulated insulin secretion after diazoxide treatment [29] is not operating when high glucose is replaced by palmitate. Hence, although the preserving effect by diazoxide on glucose-stimulated insulin release is reportedly beneficial [29], our present data suggest that this “resting” effect is less important in presence of enhanced levels of FFA (palmitate).

The present data favor the idea that excessive and longstanding generation of NO derived from iNOS is an important player in FFA-induced β-cell dysfunction after long-term culturing. This is accordance with previous data obtained in isolated islets from prediabetic Zucker diabetic fatty rats [30]. In contrast Cnop et al [31] using isolated β-cells from normal rats did not detect any iNOS mRNA expression or nitrite production in their FFA experiments. However, Cnop et al did not measure the expression and activity of iNOS protein. It is known that a great deal of endogenously generated NO is trapped within the cell by *e.g.* S-nitrosylation [32] and thus nitrite production into the incubation medium might not truly mirror the intracellular situation. Interestingly a very recent report [33] showed that both (60 min) and long-time culturing (20 h) of rat islets with the thiazolidinedione troglitazone stimulated AMP-activated protein kinase (AMPK) activity, which was associated with a decrease of glucose-stimulated insulin release after short-time and an increase after long-time treatment. Although the experimental conditions in their study were not strictly comparable to our study these data show that AMPK might be involved in the thiazolidinedione action on the β-cell. In another study with isolated β-cells [34] FFA-induced cytotoxicity was found not to be decreased but instead increased after treatment with troglitazone. Since the experimental conditions in that study was different from ours, especially with regard to the use of isolated β-cells instead of islets, the data are not comparable. Notably, however, isolated islet β-cells lack the presence of islet glucagon and glucagon-induced stimulation of β-cell adenylate cyclase is expected to suppress iNOS expression and activity [12], [13], [20].

We finally explored the involvement of the FFA receptor GPR40 in the long-term action of palmitate to induce iNOS expression and influence palmitate-stimulated insulin release. It has recently been suggested that GPR40 is necessary but not sufficient for FFA stimulation of insulin secretion *in vivo* [35]. However, these results are at variance with another recent study [6] and because the data of these experimental studies were widely different further studies are needed to finally elucidate this issue. Our results suggest that knock-down of GPR40 suppressed both palmitate-induced activity of iNOS and insulin release. Confocal microscopy confirmed the antisense inhibition of both GPR40 and iNOS expression in the β-cells. Hence, GPR40 is a major regulator of iNOS expression and insulin release during long-term exposure to palmitate. The palmitate-induced NO generation and associated suppression of glucose-stimulated insulin release is counteracted by ROZ at the GPR40 receptor, and thus, ROZ and other thiazolidinedione drugs might be beneficial for β-cell function in hyperlipidemic type 2 diabetes..

## Materials and Methods

### Animals

Female mice of the NMRI strain (B&K, Sollentuna, Sweden) weighing 25–30 g were used. They were given a standard pellet diet (B&K) and tap water *ad libitum* throughout the experiments. All animals used for preparation of pancreatic islets were killed by cervical dislocation. The experimental procedures were approved by the Ethical Committee for Animal Research at University of Lund; Sweden.

### Drugs and chemicals

Collagenase (CLS 4) was from Sigma Chemical Co (St Louis, MO, USA), HRP-conjugated goat anti-rabbit IgG was from Pierce Biotechnology, Rockford, IL, USA. Cy2-conjugated anti-mouse IgG and Cy5-conjugated anti-guinea pig IgG were from Jackson Immunoresearch Laboratories Inc, West Grove, PA, USA. Guinea pig-raised anti-insulin antibody was from Eurodiagnostica, Malmö, Sweden. Fatty acid free bovine serum albumin (BSA) was from Boehringer Mannheim, Germany. ROZ was a kind gift from Glaxo Smith-Kline, London, UK. N^G^-nitro-L-arginine methyl ester (L-NAME) was from Sigma. The insulin radioimmunoassay kits were from Diagnostika, Falkenberg, Sweden. All other chemicals were from Merck AG, (Darmstadt, Germany) or Sigma.

### Islet culturing and insulin secretion

Preparation of pancreatic islets from the mouse was performed by retrograde injection of a collagenase solution *via* the bile-pancreatic duct [14]. Islets were then isolated and hand-picked under a stereomicroscope at room temperature. After washing the islets were either used for short-term experiments and incubated as described previously [12] or used for culturing experiments. The islets were thereby cultured for 24 h in RPMI 1640 (SVA, Uppsala, Sweden) supplemented with 10% calf serum, 100 U/ml penicillin and 10 μg/ml streptomycin in the presence or absence of different test agents as indicated in the legends. After culturing the islets were preincubated for 30 min at 37°C in Krebs-Ringer bicarbonate buffer, pH 7.4, supplemented with 10 mmol/l HEPES, 0.1% BSA and 1.0 mmol/l glucose. After preincubation the buffer was changed and the islets were incubated at 1 or 20 mmol/l glucose for 60 min at 37°C unless otherwise stated. Each incubation vial contained 12 islets in 1.0 ml of buffer solution and was gassed with 95% O_2_-5% CO_2_ to obtain constant pH and oxygenation. All incubations were performed in an incubation box at 30 cycles/min. An aliquot of the medium was removed immediately after incubation and frozen for the subsequent assay of insulin. Palmitate was dissolved in ethanol (95%) with subsequent addition of stoichiometric amounts of NaOH. The stock solution was carefully evaporated under nitrogen gas. The dried residue was dissolved in water and thereafter heated to create a hot soap. The palmitate solution was stirred and fatty acid free BSA was added (10% w/v) and the pH was adjusted to 7.4 with NaOH. The solution was aliquoted and stored at –20 C. At the time of experiments, the stock solution of palmitate-BSA was diluted 1∶10 in the KRB or RPMI 1640 buffer to achieve the desired concentration of palmitate. All components of the buffer used in the experiments were prepared to be 10% more concentrated to adjust for the addition of palmitate-BSA stock solution. Under control conditions, BSA (1% w/v) was always included. The procedure has been described in detail previously [36]. In some experiments the islets were cultured for 24 h in RPMI 1640 (SVA, Uppsala, Sweden) supplemented with 10% calf serum, 100 U/ml penicillin and 10 μg/ml streptomycin in the presence or absence of either a mouse GPR40 (mGPR40) specific antisense or a nonsense morpholino oligonucleotide (Gene-Tools) at a concentration of 1.4 μM [5].

### Assay of islet NOS activities

After a culture period of 24 h, aliquots of the medium were removed for determination of insulin whereafter the islets were washed and collected in 200 μl buffer, containing 20 mmol/l HEPES, 0.5 mmol/l EDTA and 1/l mmol/l DL-dithiothreitol, and thereafter stored at −20°C. On the day of the assay, the islets were sonicated on ice and the buffer solution was enriched with 0.45 mmol/l CaCl_2_, 2 mmol l/l NADPH, 25 U/ml calmodulin, and 0.2 mmol/l L-arginine. For the determination of iNOS activity both Ca^2+^ and calmodulin were omitted. The homogenate was incubated at 37°C under constant air bubbling with air, 1.0 ml/min for 2 h. Aliquots of the incubated homogenate (200 μl) were then passed through an 1 ml Amprep CBA cation-exchange column for determination of L-citrulline by high performance liquid chromatography (HPLC). The method has been described in detail [18]. Since L-citrulline and NO are generated in equimolar amounts, and since L-citrulline is stable whereas NO is not, L-citrulline is the preferred parameter when measuring NO production. Protein concentration was determined according to Bradford [37] on samples from the original homogenate.

### Immunofluorescence and confocal microscopy

Culturing of the freshly isolated islets (24 h) in the presence of different agents were performed as stated above for the assay of islet NOS activities. An aliquot of the medium was removed for determination of insulin. The islets were then washed (3 times) and fixed with 4% formaldehyde, permeabilized with 5% Triton X-100, and unspecific sites were blocked with 5% Normal Donkey Serum (Jackson Immunoresearch Laboratories Inc, West Grove, PA). iNOS was detected with a rabbit-raised polyclonal anti-iNOS antibody (StressGen Biotechnologies Corp, Victoria, BC, Canada) (1∶100) in combination with Cy2-conjugated anti-rabbit IgG (Jackson Immunoresearch Laboratories Inc, West Grove, PA) (1∶150). For staining of insulin, islets were incubated with a guinea pig-raised anti-insulin antibody (Eurodiagnostica, Malmö, Sweden) (1∶1000) followed by an incubation with a Cy5-conjugated anti-guinea pig IgG antibody (Jackson Immunoresearch Laboratories Inc, West Grove, PA) (1∶150). For scoring of iNOS positive cells in islets multiple fields for each section were analysed under blind conditions. The mean fluorescence intensity of cellular iNOS was quantified using Zeiss LSM 5 analysis software. The methodology for detection of mouse GPR40 has recently been described [21]. Briefly, a polyclonal antibody (1∶100) in combination with Cy2-conjugated anti-mouse IgG (1∶150) were used. The receptor specific antibody was raised in rabbit against the C-terminal peptide: NH2-CVTRTQRGTIQK-COOH. The fluorescence was visualized with a Zeiss LSM510 confocal microscope by sequentially scanning at (excitation/emission) 488/505–530 nm (Cy2) and 633/>650 nm (Cy5).

### Cell culture and transfection

A subclone of the MIN6 cell line, MIN6c4, was grown in Dulbecco's modified Eagle's medium (DMEM) with Glutamax-1 (Invitrogen, Paisley, UK) supplemented with 15% heat-inactivated FBS (Invitrogen), 60 μM β-mercaptoethanol, 50 U/ml penicillin, and 50 μg/ml streptomycin. HEK293 cells were grown in DMEM with Glutamax-1 supplemented with 3% FBS, 50 units/ml penicillin and 50 μg/ml streptomycin. All cells were maintained in a 37°C incubator with 7% CO2. The mouse GPR40 ORF (Genbank accession number AB095745) was amplified with PCR (forward primer, 5′ GCCAAGCTTACCATGGACCTGCCCCCACAGCTCTCCTTCG 3′; reverse primer, 5′ GGCGAATTCCTACTTCTGAATTGTTCCTCTTTGAGTC 3′), subcloned into the pEAK12 expression vector (Edge BioSystems, Gaithersburg, MD), and then transfected into HEK293 cells using Lipofectamine 2000 (Invitrogen), according to the manufacturer's instructions. Total time of transfection was 6 h and the cells were assayed 48 h later.

### PI hydrolysis

Receptor activation by FFA was assayed in the HEK293 and MIN6c4 cells by measuring hydrolysis of phosphatidyl inositol (PI) [38]. Briefly, MIN6c4 cells (approximately 100000 cells per well) were pre-loaded with myo-[3H]inositol (Perkin Elmer, Boston, MA) for 16–20 h and then thoroughly washed and incubated in the KRB-buffer at 8.3 mmol/l glucose in the absence or presence of palmitate (1 mmol/l)±ROZ (at different concentrations) for 30 min. After the incubation the cells were lysed with formic acid on ice and the inositol phosphates were isolated using anion exchange chromatography. The PI hydrolysis were expressed as PI hydrolysis per well. The coefficient of variation (interassay differences) was 7% for MIN6c4 cells.

### Antisense intervention

Isolated islets (250 islets/vial) were incubated for 30 min with palmitate (1 mmol/l) in KRB solution. After washing islets were then cultured for 48 h in the absence or presence of 1.4 μmol/l of M40 morpholino oligonucleotide. At day 2 (after 24 h culture) palmitate (1 mmol/l) was added to the culture medium and the islets were cultured for an additional period of 24 h. A nonspecific random-sequence morpholino was used as control [5]. The morpholino oligonucleotide was loaded into the islets using the Gene-Tools special delivery system according to the manufacturers instructions.

### Determination of insulin

Insulin secretion and insulin content of the islets were determined by radioimmunoassay [39].

### Statistics

Results were expressed as means±s.e.m. The level of significance for the difference between sets of data was assessed using Student's unpaired *t*-test or analysis of variance followed by Tukey-Kramer's test whenever appropriate. P<0.05 was considered statistically significant.
