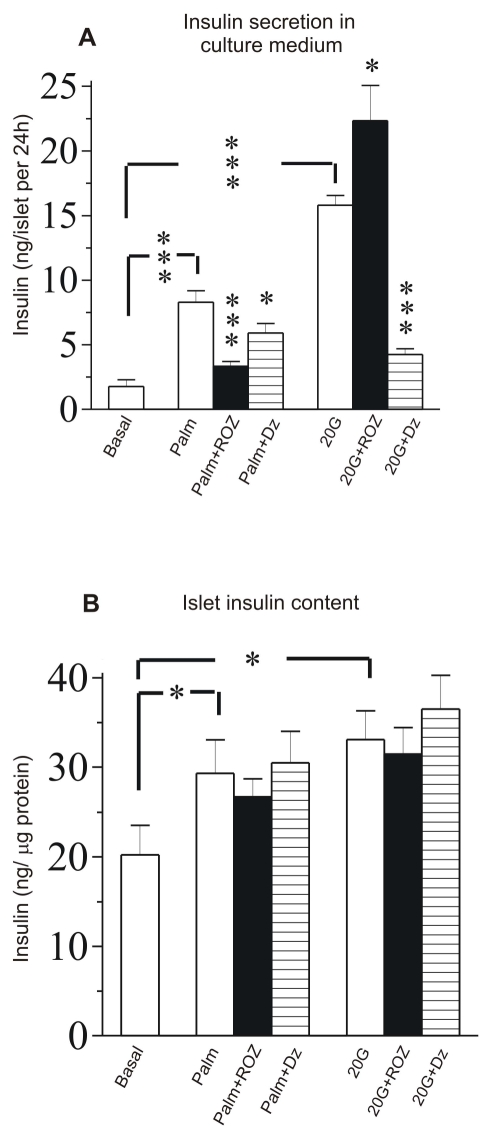# Correction: Palmitate-Induced β-Cell Dysfunction Is Associated with Excessive NO Production and Is Reversed by Thiazolidinedione-Mediated Inhibition of GPR40 Transduction Mechanisms

**DOI:** 10.1371/annotation/21d1a1f6-35e9-4935-a8d0-387c057f1469

**Published:** 2008-05-20

**Authors:** Sandra Meidute Abaraviciene, Ingmar Lundquist, Juris Galvanovskis, Erik Flodgren, Björn Olde, Albert Salehi

There was an error in the y-axis label of Figure 2B. The corrected figure is available here:

**Figure:**