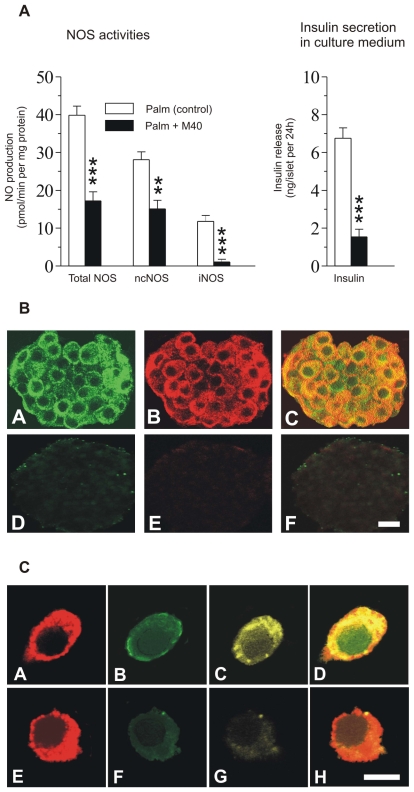# Correction: Palmitate-Induced β-Cell Dysfunction Is Associated with Excessive NO Production and Is Reversed by Thiazolidinedione-Mediated Inhibition of GPR40 Transduction Mechanisms

**DOI:** 10.1371/annotation/682082fc-c500-414b-9f57-f9420c00c57e

**Published:** 2008-05-20

**Authors:** Sandra Meidute Abaraviciene, Ingmar Lundquist, Juris Galvanovskis, Erik Flodgren, Björn Olde, Albert Salehi

There was an error in part D of Figure 5C. Part D was incorrectly a duplicate of part C. The corrected figure is available here:

**Figure:**